# Exploring mito-nuclear genetic factors in Leber's hereditary optic neuropathy: insights from comprehensive profiling of unique cases

**DOI:** 10.17179/excli2023-6297

**Published:** 2023-10-09

**Authors:** Prakash Chermakani, Poigaialwar Gowri, Shanmugam Mahesh Kumar, Periasamy Sundaresan

**Affiliations:** 1 Department of Molecular Genetics, Aravind Medical Research Foundation, Madurai, Tamil Nadu, India; 2 Department of Molecular Biology, Aravind Medical Research Foundation - Affiliated to Alagappa University, Karaikudi, Tamil Nadu, India; 3 Neuro Ophthalmology Clinic, Aravind Eye Hospital, Madurai, Tamil Nadu, India

**Keywords:** mitochondrial complex I disorder, retinoganglion degeneration, mito-nuclear genetic factors, arLHON, optic atrophy and vision loss

## Abstract

Leber's hereditary optic neuropathy (LHON) is a mitochondrial complex I disorder and causes inexorable painless vision loss. Recent studies from India reported that a significant proportion of LHON patients lack primary mitochondrial DNA mutations, suggesting that alternative genetic factors contribute to disease development. Therefore, this study investigated the genetic profile of LHON-affected individuals in order to understand the role of mito-nuclear genetic factors in LHON. A total of thirty probands displaying symptoms consistent with LHON have undergone whole mitochondrial and whole exome sequencing. Interestingly, whole mtDNA sequencing revealed primary mtDNA mutations in 30 % of the probands (n=9), secondary mtDNA mutations in 40 % of the probands (n=12) and no mitochondrial changes in 30 % of individuals (n=9). Further, WES analysis determined pathogenic mutations in 11 different nuclear genes, especially in cases with secondary mtDNA mutations (n=6) or no mtDNA mutations (n=6). These findings provide valuable insight into LHON genetic predisposition, particularly in cases lacking primary mtDNA mutations.

See also Figure 1(Fig. 1).

## Abbreviations

ACMG American College of Medical Genetics and Genomics

ADOA autosomal dominant optic atrophy

arLHON autosomal recessive LHON

BCVA Best corrected visual acuity

DNAJC30 DnaJ Heat Shock Protein Family (Hsp40) Member C30

ETC Electron transport chain

LHON Leber's hereditary optic neuropathy

LS Leigh syndrome

MC1DN Mitochondrial complex I deficiency nuclear types

mtDNA mitochondrial DNA

MTFMT Mitochondrial Methionyl-tRNA formyl transferase

NDUFA3 NADH: Ubiquinone Oxidoreductase Subunit A3

NDUFS2 NADH: Ubiquinone Oxidoreductase Core Subunit S2

NDUFS7 NADH: Ubiquinone Oxidoreductase Core Subunit S7

NDUFV1 NADH: Ubiquinone Oxidoreductase Core Subunit V1

PDSS1 Decaprenyl diphosphate synthase subunit 1

RGCs Retino ganglion cells

RNFL Retinal nerve fibre layer

SLC25A3 Solute Carrier Family 25 Member 3

SLC25A46 Solute Carrier Family 25 Member 46

WES Whole exome sequencing

## Introduction

In the late 1850s, Albrecht von Graefe observed acute central vision loss among young males (Von Graefe, 1858[31]). The clinical basis of this disease was first articulated by Theoder Leber in 1871 and it is termed Leber's hereditary optic neuropathy (LHON) (Leber, 1871[15]). Until 1972, LHON was presumed to be inherited X-linked. Later, Erickson suggested that mitochondrial dysfunction was responsible for the non-Mendelian transmission (Erickson, 1972[4]). Subsequently, D. C. Wallace confirmed that this disease was inherited maternally by identifying primary mutations in mitochondrial genes (MT-ND1, MT-ND4, MT-ND6) coding for electron transport complex I (ETC). These mutations are referred to as primary mutations (Wallace et al., 1988[32]). 

Clinically, LHON distinguishes itself from other hereditary optic atrophies by its progressive loss of central vision and acute degeneration of retinal ganglion cells (RGCs) (Neuhann and Rautenstrauss, 2013[23]). These cells have specialized functions to conduct visual signals from the eye to the brain via the optic nerve to form an image. However, the precise cause of RGCs degeneration remains unknown. Several hypotheses suggested that vision loss may be caused by the energy demands of the eyes.

Indeed, LHON often manifested with gender bias and partial penetrance within families. However, it is further complicated by the involvement of nuclear genes and other environmental factors contributing to disease heterogeneity. Of late, several mitochondrial disorders have been ascertained to be caused by mutations in nuclear genes, posing a major diagnostic challenge due to overlapping clinical symptoms (Yu-Wai-Man et al., 2016[36]). 

According to our previous five-year hospital-based study, only 43.6 % of LHON patients had primary mutations (Gowri et al., 2020[7]). Besides, a comprehensive analysis of 100 South Indian LHON patients using whole mitochondrial DNA (mtDNA) sequencing also revealed 42 % of cases harbor primary mutations, while 13 % had secondary mutations (Gowri et al., 2022[8]). Another research group from South India found that only 29.4 % of individuals displayed primary mutation (Sundaramurthy et al., 2021[29]). The prevalence of primary mutations in LHON patients from India is predicted to be 29.4-43.6 % lower than the global average (95 %). Consequently, it is speculated that the remaining 60 % of patients might have either secondary mtDNA mutations or mutations in nuclear genes regulating mitochondrial dynamics. Therefore, the present study aimed to investigate the genetic basis of thirty distinct LHON cases through comprehensive sequencing to identify mito-nuclear genetic factors involved in the disease pathogenesis. Our findings highlight the significance of nuclear gene involvement in cases of LHON exhibiting secondary mutations or inconclusive mtDNA mutations.

## Materials and Methods

Thirty patients with LHON-like optic neuropathy were clinically diagnosed at the neuro-ophthalmology clinic, Aravind Eye Care System in Madurai, India. A comprehensive ophthalmic examination was conducted on all participants, including an assessment of best-corrected visual acuity (BCVA), visual field test and fundus examination. The study participants were selected based on the following criteria includes patients experiencing sudden painless central vision loss, exhibiting optic disc hyperemia, temporal pallor, thickening of the retinal nerve fiber layer (RNFL), vessel tortuosity, and peripapillary telangiectasias. Informed consent was obtained from all study participants, including minors, or their legal guardians. Genealogical information was collected from all family members (Supplementary Figure 1). The peripheral blood samples were obtained using an EDTA vacutainer and genomic DNA extraction was performed using a modified salting-out method (Miller et al., 1988[18]). The study strictly adhered to the principles of the Helsinki Declaration and received approval from the Institutional Ethics Committee of Aravind Eye Hospital (AEH), Madurai, India.

### Mitogenome sequencing

A total of twenty-four different primers were used to amplify the whole human mitochondrial genome (~16.5 kb) of the control and the proband and then it was sequenced through Sanger sequencing (Gowri et al., 2020[7]). Furthermore, haplogroup analysis was performed on all thirty study participants using haplogrep 2.0 software to determine their maternal lineage's geographic origin.

### Whole exome sequencing

All study participants underwent WES using Agilent Sure Select XT Human All Exon V5. DNA libraries were prepared and the samples were pooled and loaded in equimolar concentrations on the Illumina Hi Seq X10 platform for massive parallel paired-end DNA sequencing for 300 cycles to generate 2X150 bp sequence reads at 80-100X depth.

### Variant calling and filtering

After sequencing, the raw FASTQ file was subjected to adaptor trimming, and then aligned with human reference genome build hg19/GRCh37 using Sention's version of the BWA aligner. Further, PCR duplicates were removed using Sentieon's Picard tool. Subsequently, variant calling was carried out using Sentieon's GATK Haplotype Caller and Unified Genotyper, and the variants were annotated using VariMAT. Primarily, the variants were prioritized based on their minor allele frequency, with the cut-off value <0.01 (1 %) in the following population databases including 1000 genome, ESP, ExAC, and gnomAD. Further, 1136 nuclear genes were selected to emphasize mitochondrial protein localization across 14 tissues based on the Mitocarta 3.0 database. Subsequently, these variants including (nonsynonymous missense variants, nonsense variants, splice variants and frameshift indels) can be narrowed down on the basis of their evolutionary conservation score and pathogenicity by using the following tools Polyphen2, SIFT, Mutation Taster, FATHMM, LRT, GERP and CADD (Stelzer et al., 2016[27]). All the variants identified in this study were classified based on the five categories outlined in the American College of Medical Genetics and Genomics (ACMG) guidelines: pathogenic, likely pathogenic, uncertain significance, likely benign, and benign. 

### Statistical analysis

The association between nuclear gene mutation and mitochondrial mutation was assessed using Fisher's Exact Test. Also, the association of primary mutation and secondary mutation with nuclear genes was analyzed using Fisher's Exact test. Furthermore, one-way ANOVA was conducted on all three groups to analyze nuclear gene involvement. In hypothesis testing, a significance level of p < 0.05 was considered statistically significant. The estimation of unknown parameters was described using a 95 % confidence interval (CI).

## Results

**Figure 2:**
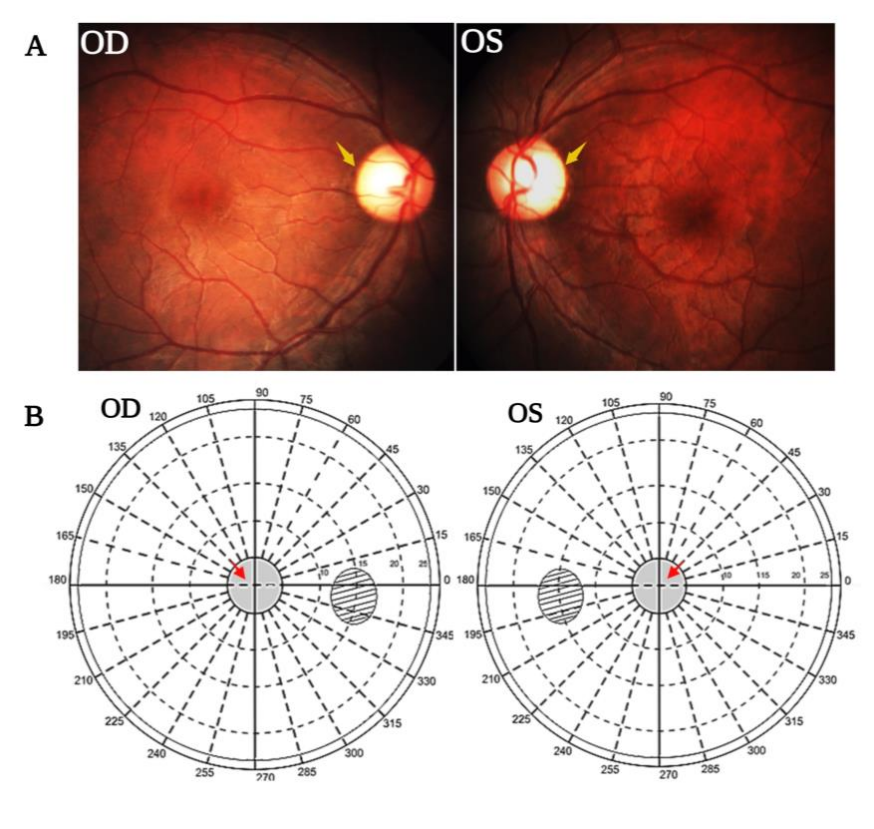
LHON patient fundus appearance and central visual field defects A: Optic disc appearance of an LHON patient displayed temporal pallor in both eyes (indicated in yellow arrow). OD represents the right eye; OS represents the left eye. B: Central visual field of LHON patient with central scotoma (defective fovea) represented by the gray shaded region within a 30° radius of both eyes. The red arrow indicates the location of the scotoma near the blind spot.

This study included 30 unrelated probands suspected of LHON, pertaining to the South Indian ethnic group. The median age of disease presentation was 20.5 years (ranging from 10 to 35). Of these, 3 % (n=1) in the first decade, 47 % (n=14) in their second decade, 43 % (n=13) in their third decade and 7 % (n=2) in their fourth decade. The patients with defective central vision exhibit temporal pallor (paleness in the temporal region of the optic disc), which irreversibly affects RGC's axons (Figure 2(Fig. 2)). Furthermore, the visual impairment of study subjects was classified based on their BCVA as follows mild (6/10-6/18) (n= 5, 17 %), moderate (6/24-6/48) (n= 4, 13 %), severe (6/60-3/60) (n= 14, 47 %), profound (2/60) (n= 3, 10 %) and near blindness (1/60 or less) (n= 4, 13 %). Table 1(Tab. 1) lists the complete ophthalmic clinical characteristics of all study participants.

**Figure 3:**
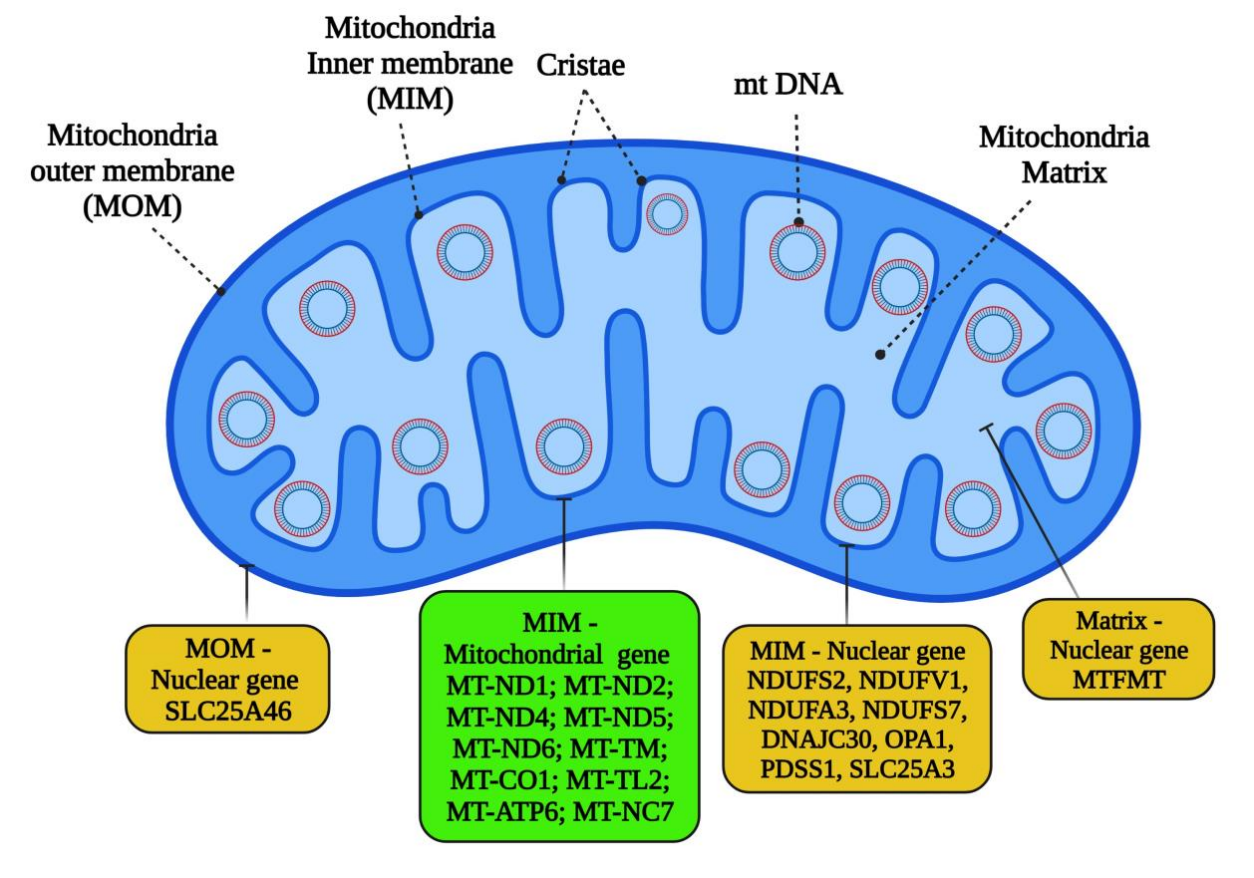
Mito-nuclear genes linked to LHON. This list summarizes the mutant genes identified in the study. The mtDNA-encoded genes highlighted in green and the nuclear DNA-encoded genes highlighted in yellow are associated with LHON. The genes are categorized based on its mitochondrial protein localization in the mitochondrial matrix (1 gene), the outer membrane (1 gene), and the inner membrane (18 genes). Detailed information about the genes and their phenotypes is represented in Table 1 and Table 2.

The whole mtDNA sequencing result showed about 70 % of probands had mutations in their mitochondrial genome. Based on these findings, the probands were further subdivided into three groups. Probands in Group I had primary mtDNA mutations (n=9, 30 %), Group II probands harbored secondary mtDNA mutations (n=12, 40 %) and probands in Group III did not have any mtDNA mutations (n=9, 30 %). The schematic representation of the mito-nuclear genetic variants is shown in (Figure 3(Fig. 3)). A comprehensive list of the pathogenic variants found in LHON patients is provided in Table 2(Tab. 2). 

In addition, haplogrep analysis identified five different haplogroups: M (n=23), U (n=4), N (n=1), P (n=1), and R (n=1). Furthermore, WES results displayed pathogenic nuclear gene mutations in 40 % of probands. Remarkably, most of the nuclear genes ascribed in our study were associated with mitochondrial complex I deficiency nuclear types (MC1DN). Overall, the analysis of nuclear gene involved in the study cases resulted in a p-value of 0.102, suggesting a marginal level of significance. 

Ultimately, the comprehensive mitochondrial variant list for all study subjects is provided in Supplementary Table 2. Moreover, the FASTQ files comprising the WES data for the thirty study participants have been deposited in the National Center for Biotechnology Information - Sequence Read Archive (NCBI-SRA) database. These submissions are categorized with the following project IDs: PRJNA1013110 (Sample ID 1-10), PRJNA1013291 (Sample ID 11-20), and PRJNA1013221 (Sample ID 21-30).

### Distribution of mito-nuclear genetic variations within the study groups

#### Group I:

A total of nine individuals were found to have primary mutations in the mitochondrial genome. These mutations were encompassed in m.3460G>A/Mt-ND1 (n=2), m.11778G> A/Mt-ND4 (n=3), m.14484T>C/Mt-ND6 (n=3) and a rare primary mutation (m.4171C > A) in MT-ND1. Additionally, three individuals had co-occurrence of more than one mitochondrial variant, specifically m.4216T> C/MT-ND1, m.12033A>G/MT-ND4 and m.13708G>A/MT-ND5. It is worth noting that WES analysis from this group did not reveal any pathogenic variants linked to LHON. A summary of all the sequencing chromatograms from group I is shown in Supplementary Figures 2 and 2a. Among the nine probands, four individuals had a family history of LHON. Specifically, the male-to-female ratio was 8:1, highlighting a significant male preponderance (Supplementary Table 1).

#### Group II:

Twelve individuals had secondary mtDNA mutations associated with LHON. Among them, six individuals carried a single mitochondrial variant: m.13708G>A in MT-ND5, m.9139G>A in ATP6, m.4454T>C in MT-TM, m.11544T>A in MT-ND4, m.7859G>A in MT-CO2, and m.4216T>C in MT-ND1. In addition, one proband exhibited 9 bp deletion in NC-7 gene. Moreover, five probands had two mitochondrial variants such as (m.4842A>G/MT-ND2 and m.12308A> G/MT-TL2); (m.8950G>A/MT-ATP6 and m.11696G>A/MT-ND4); (m.4216T>C/MT-ND1 and m.7444G>A/MT-CO1); (m.13708G>A/MT-ND5 and m.15927G> A/MT-TT); (m.4216T>C/MT-ND1 and m.8573G>A/MT-ATP6). Noteworthy, only one proband in this group had a family history and the male-to-female ratio was 1.4:1. Similarly, the proband with secondary mtDNA mutations alone exhibits a male-to-female ratio of 2:1. In contrast, probands with both nuclear gene mutations and secondary mutations exhibit a male-to-female ratio of 1:1 (Supplementary Table 1). 

Furthermore, WES results indicated 50 % of the probands in group II had mutations in nuclear genes NDUFS2, NDUFS7, OPA1, MTFMT, PDSS1, and MYOC. Notably, mutations in NDUFS2, NDUFS7, and MTFMT were strongly associated with mitochondrial complex I deficiency nuclear types. A proband was identified with compound heterozygous mutation (c.G812A) in exon 8 and (c.T1220G) exon 12 of the NADH: Ubiquinone Oxidoreductase Core Subunit S2 (NDUFS2) gene. Likewise, in two other probands, one exhibited a homozygous transition missense variant (c.C431T) in the exon 6 of NADH: Ubiquinone Oxidoreductase Core Subunit S7 (NDUFS7) gene, whereas the other displayed a mutation (c.T476C) in the exon 3 of Mitochondrial Methionyl-tRNA formyl transferase (MTFMT) gene. Additionally, a proband was detected with two-base pair deletion (c.526_527del) in the exon 5 of OPA1 gene which regulates mitochondrial dynamics as a dynamin-like GTPase. Besides, a proband was found with a homozygous transversion mutation (c.A854G) in the exon 9 of the Decaprenyl diphosphate synthase subunit 1 (PDSS1) gene. Interestingly, a proband was diagnosed with defective central vision resembling LHON and had been treated with the anti-tuberculosis drug ethambutol for an extended period, leading to toxic neuropathy. Correspondingly, WES analysis of this proband revealed a point mutation (c.G144T) in the exon 1 of the MYOC gene. In group II, a noteworthy number of probands exhibited nuclear gene mutations in comparison to group I, with a p-value of 0.019. Supplementary Figures 3 and 4 illustrate the chromatograms of the mito-nuclear genetic variants identified in group II.

#### Group III:

The study probands (n=9) in group III did not harbor any mtDNA mutations. However, WES analysis revealed mutations in nuclear genes across six probands (67 %). Mutations were found in the following genes encoding NDUFV1, NDUFA3, DNAJC30, SLC25A3, and SLC25A46. In two probands, a homozygous missense mutation (c.C1156T) was detected in exon 8 of the NADH: Ubiquinone Oxidoreductase Core Subunit V1 (NDUFV1) gene. Similarly, a proband exhibited a homozygous missense variant (c.C148A) in exon 3 of the accessory subunit NADH: Ubiquinone Oxidoreductase Subunit A3 (NDUFA3) gene, which is involved in electron transport complex I assembly. Furthermore, a proband displayed a homozygous nonsense mutation (c.C118T) in exon 1 of the DnaJ Heat Shock Protein Family (Hsp40) Member C30 (DNAJC30) gene. Afterward, two probands, of which one showed a homozygous missense variant (c.G745C) in the exon 7 of Solute Carrier Family 25 Member 3 (SLC25A3) gene, while the other displayed a mutation (c.A670G) in the exon 7 of Solute Carrier Family 25 Member 46 (SLC25A46) gene. Consequently, 33 % of the probands are genetically unresolved, which further emphasizes the importance of whole genome sequencing to understand the pathogenesis of LHON. A chromatogram of nuclear genetic variants identified in group III is shown in Supplementary Figure 5.

## Discussion

This study aimed to investigate the genetic profiles of LHON probands to decipher the key mito-nuclear genetic factors and their implications in disease pathogenesis. The median age of the probands involved in this study was approximately 20 years, which was consistent with previous LHON studies (Poincenot et al., 2020[25]). A number of studies indicate that probands with primary mutations in mtDNA are more likely to develop LHON (Yu-Wai-Man et al., 2002[35]). In the current study, nine probands had primary mutations in their mtDNA, of which three probands exhibited co-occurrences of more than one mitochondrial variant in the ETC I subunit. According to the previous report, primary mtDNA mutations that coexist with secondary mtDNA mutations were found to have a synergistic effect (Jancic et al., 2020[10]). For instance, mutations such as (m.11778G>A/MT-ND4 & m.4216T>C/MT-ND1), and (m.14484T>C/MT-ND6 & m.13708 G>A/MT-ND5) have been found to contribute to the development of LHON in the J haplogroup (Torroni et al., 1997[30]). Furthermore, an Italian study demonstrated that the combination of mtDNA mutations in genes m.12033A>G/MT-ND4 and m.14258G>A/MT-ND6 affects proton pumping in the ETC and causes LHON (Caporali et al., 2018[3]). Additionally, our study suggests that m.12033A>G and m.11778G>A mutations in the MT-ND4 gene may have synergistic effects on LHON. Interestingly, the WES of probands from group I did not reveal any pathogenic nuclear genetic variants associated with LHON. These findings highlighted that primary mtDNA mutations are more likely to cause LHON in group I probands than nuclear genetic variants.

A substantial proportion of the probands in Group II displayed mutations in other modifier genes related to OXPHOS dysfunction. Among them, six probands were found to have single-point mutations in mtDNA. The identified mutations were as follows: m.13708G>A in MT-ND5, m.9139G>A in ATP6, m.4454T>C in MT-TM, m.11544T>A in MT-ND4, m.7859G>A in MT-CO2, and m.4216T>C in MT-ND1. Mutations in mtDNA m.4216T>C in MT-ND1 and m.13708G>A in MT-ND5, which belong to the M haplogroup, has been extensively documented and linked to LHON. Furthermore, a meta-analysis of these LHON-associated variants indicated that secondary mutations in these genes, either alone or in combination with primary mutations in the M and R haplogroups, increase the risk of LHON progression (Jha et al., 2021[11]). Despite these findings, one individual in the present study harbors a mutation in m.13708G>A of the MT-ND5 gene, which belongs to the U haplogroup. Nevertheless, the functional impact of this mutation in the U haplogroup warrants a cybrid model. Another individual from this study exhibited m.9139G>A mutation in the ATP6 gene encoding ETC V subunit. Apulian cohort studies showed that this variant combined with primary mutations in the T haplogroup had a synergistic effect (La Morgia et al., 2008[13]). Moreover, our previous study also demonstrated that mutations in ATP6 (m.9139G>A) and MT-ND4 (m.11544T>A) are responsible for 1 % of LHON cases in the South Indian cohort (Gowri et al., 2022[8]). Additionally, a mitochondrial variant m.4454T>C in the MT-TM gene was predicted to be a possible contributor to mitochondrial dysfunction (Jha et al., 2021[11]). Interestingly, one proband had a novel 9-bp deletion in the NC-7 gene, but its functional significance to LHON pathogenesis is unclear. However, a Tunisian study described that mitochondrial non-coding region NC7 gene triplication causes mitochondrial neuromuscular disorders (Mkaouar-Rebai et al., 2016[19]). The present study also discussed a rare case with mutations in two mitochondrial genes (MT-ND2 (m.4842A>G) and MT-TL2 (m.12308A>G). Consequently, it is reported that individuals without primary mtDNA mutations carried a point mutation (m.12308A>G) in the MT-TL2 gene and exhibited a significantly reduced mitochondrial copy number with low penetrance among the Chinese Han population (Wang et al., 2020[33]). Moreover, the Clinvar database demonstrates that the mutation m.4842A>G in the MT-ND2 gene is linked with Leigh syndrome (LS) (Landrum et al., 2014[14]).

Strikingly, two probands in Group II harbored one mitochondrial variant (m.4216T>C in MT-ND1 and m.7859G>A in MT-CO2), along with a nuclear genetic variant in the NDUFS2 gene and OPA1 gene, respectively. The NDUFS2 subunit is located in the Q-module of an L-shaped ETC I subunit in an inner mitochondrial membrane. It transfers electrons to ubiquinone that pumps the proton in P-module (Kashani-Poor et al., 2001[12]). A mutation in the NDUFS2 gene is associated with mitochondrial complex I deficiency nuclear type 6 (Hamosh et al., 2005[9]). It has been reported that compound heterozygous mutations of the NDUFS2 gene are associated with non-syndromic arLHON (autosomal recessive LHON). A French research group studied the functional consequences of compound heterozygous variants of NDUFS2 analogs using a yeast model. The mutant p. His57Cys of NDUFS2 did not significantly alter complex I of mitochondrial membranes, while the mutant p. Tyr311Cys lacked complex I activity (Gerber et al., 2017[5]). The present study shows that a proband with a distinct combination of a novel pathogenic compound heterozygous mutations in the NDUFS2 gene. Alongside, the proband was also found to harbor a mitochondrial variant (m.4216T>C) known to disrupt the electron transfer process in mitochondria. In previous studies, mutations in the OPA1 gene had been associated with autosomal dominant optic atrophy (ADOA) (Pesch et al., 2001[24]). The current investigation observed a proband with a frameshift mutation in the OPA1 gene. This specific mutation may impair mitochondrial fusion, resulting in mitochondrial dysfunction. Furthermore, a proband was identified with two mitochondrial variants (m.8950G> A/MT-ATP6 and m.11696G>A/MT-ND4), as well as a nuclear mutation (c.C431T) in exon 6 of the NDUFS7 gene. According to Polish research, mitochondrial variant m.8950G>A/MT-ATP6 severely impairs ATP production (Baranowska et al., 2023[2]). Similarly, it has been shown that the m.11696G>A mutation in the MT-ND4 gene exhibited higher penetrance in the Chinese Han population, acting synergistically with the primary mutation (Xie et al., 2017[34]). A mutation in the NDUFS7 gene, another important core subunit of ETC I, located inside the Q-module, has been linked to LS. Notably, a Tunisian patient with an atypical Leigh phenotype was reported to have a homozygous missense mutation in NDUFS7, which altered arginine 145 to histidine (Lebon et al., 2007[16]). In this present study, a proband exhibited an amino acid change from proline 144 to lysine in the NDUFS7 gene; however, he did not display any other neuromuscular symptoms or lactic acidosis typically associated with LS. Likewise, a mutation in the MTFMT gene has also been associated with combined oxidative phosphorylation deficiency, particularly complex I and IV, as well as isolated complex I deficiency. It was previously found that the MTFMT gene has a compound heterozygous mutation in exons 3 and 9 leading to reduced activity of complex I and IV (Neeve et al., 2013[22]). It was unusual that the proband in the present study did not have previous symptoms of neuromuscular disorders, hypotonia, or ataxia, but had signs of optic atrophy. Therefore, LHON was presumed to be caused by a mutation in exon 3 of the MTFMT gene, along with the mitochondrial variants (m.4216 T>C and m.7444 G>A). Furthermore, very few reports suggest a strong association between mutations in PDSS1 and optic atrophy or sensorineural deafness. A proband in this study was found to carry a novel missense variant (c. A854G) in exon 9 of the PDSS1 gene, along with secondary mtDNA mutations (m.13708 G>A and m.15927 G>A). Apparently, these mutations disrupted the biosynthesis of coQ-10 and reduced cellular energy (Nardecchia et al., 2021[21]). It is noteworthy that the proband did not display any other hearing, cardiac, or kidney impairments. Eventually, a proband was found to carry a mutation (c. G144T) in exon 1 of the MYOC gene, which is associated with primary open-angle glaucoma (Mukhopadhyay et al., 2002[20]). It is important to note that this patient also lost his central vision due to the excessive intake of anti-tuberculosis medicine ethambutol. Overall, group II results emphasize although mtDNA mutations are primarily responsible for LHON, substantial evidence suggests that nuclear genes may also play an important role in disease progression. However, further research is needed to explore the impact of nuclear genes role in LHON severity.

In group III, approximately 67 % of probands had mutations exclusively in nuclear genes regulating mitochondria. It is interesting to note that two individuals in this group harbor a mutation (c.C1156T) in exon 8 of the NDUFV1 gene. The NDUFV1 gene is also located in the Q-module of the L-shaped structure of the ETC I subunit. Previous reports have predicted that the same missense variant in the NDUFV1 gene could impact electron shuttling, which is crucial for efficient energy production. Moreover, it was associated with a childhood disorder LS, typically characterized by progressive neurological impairment (Srivastava et al., 2018[26]). Intriguingly, neither of these individuals exhibited the classic Leigh phenotype. Instead, they displayed clinical features that resembled LHON, suggesting that NDUFV1 missense variants have different impacts on affected individuals, resulting in distinct clinical manifestations. Additionally, a proband also carried a novel missense variant (c.C148A) in exon 3 of the NDUFA3 gene. NDUFA3, a supernumerary subunit, plays a critical role in the assembly of the Q-module within complex I. It is worth noting that as of yet NDUFA3 gene hasn't been reported to have any mutations. However, a complete deletion of NDUFA3 has been reported to be associated with retinitis pigmentosa without any sign of complex I deficiency (Golovleva et al., 2010[6]). Remarkably, this is the first study to associate a mutation in the NDUFA3 gene as the cause of acute optic atrophy mimicking LHON. However, it requires further investigation of the functional implications of this mutation on ATP respiration levels. This study also identified an individual carrying a novel nonsense mutation in the DNAJC30 gene, which results in a premature stop codon associated with arLHON. The DNAJC30 protein chaperone facilitates the replacement of damaged subunit within complex I and is associated with arLHON. Earlier studies have shown that biallelic knockout of DNAJC30 in HEK293 cells and patient-derived fibroblasts result in significant disruptions of ATP turnover, particularly within the complex I N-module. This suggests dysfunctional DNAJC30 may impair the proper replacement of damaged subunit in complex I, possibly contributing to arLHON (Stenton et al., 2021[28]). Further, a proband also had a putative mutation (c. A670G) in exon 7 of the SLC25A46 gene. Specifically, the SLC25A46 gene facilitates mitochondrial/endoplasmic reticulum lipid transfer and mitochondrial fission, and it plays a significant role in mitochondrial dynamics. Mutations in SLC25A46 are often associated with LS (Abrams et al., 2015[1]). However, it should be noted that the proband in our study did not display spasticity despite the absence of peripheral neuropathy and cerebellar involvement. Despite the diverse clinical manifestations associated with SLC25A46 mutations, these findings emphasize the need for comprehensive genetic testing in patients with similar symptoms. Likewise, another proband exhibited a novel mutation (c. G745C) in exon 7 of the SLC25A3 gene. Mutations in SLC25A3 are considered a novel disorder that affects oxidative phosphorylation (OXPHOS). This gene transports inorganic phosphate into the mitochondrial matrix, a crucial step in aerobic ATP synthesis. Previously, a homozygous mutation (c.215G>A) in SLC25A3 has been associated with ATP synthesis deficiency in muscle biopsy (Mayr et al., 2007[17]). Despite this, our proband did not show signs of hypertrophic cardiomyopathy or muscular hypotonia. The findings indicate that mutations in SLC25A3 may result in a wide range of clinical presentations and highlight the importance of further exploration of the phenotypic spectrum of this disorder. About 33 % of the probands in group III remained genetically unresolved and neither mtDNA nor nuclear gene mutations. This suggests that other underlying genetic and environmental factors may also play a role in LHON development, emphasizing the need for whole genome sequencing and considering other potential factors to decode the LHON pathogenesis.

## Conclusion

The present work sheds light on the significance of mito-nuclear genetic involvement in LHON disease pathogenesis. Furthermore, this study also emphasizes the importance of comprehensive genetic evaluations, including both mtDNA and nuclear gene analyses, in the diagnosis and management of LHON. Early diagnostic intervention may preserve residual vision and prevent further optic nerve damage. In addition, the mutations found in nuclear genes such as NDUFS2, OPA1, NDUFS7, MTFMT, PDSS1, NDUFV1, NDUFA3, DNAJC30, SLC25A46, SLC25A3 and MYOC extend our understanding of the genetic landscape of LHON. However, the functional impact of mito-nuclear interactions on LHON will be investigated in the near future, to provide further insight into the potential therapeutic targets for this vision-threatening disorder.

## Declaration

### Acknowledgments

Prakash Chermakani acknowledges the Indian Council of Medical Research for Senior Research Fellowship under the discipline Gen/BMS Project ID 2020-8159.

### Funding

This research work was supported by a SERB CRG grant: CRG/2022/000926.

### Conflict of interest

The authors declare no conflict of interest.

## Supplementary Material

Supplementary information

## Figures and Tables

**Table 1:**
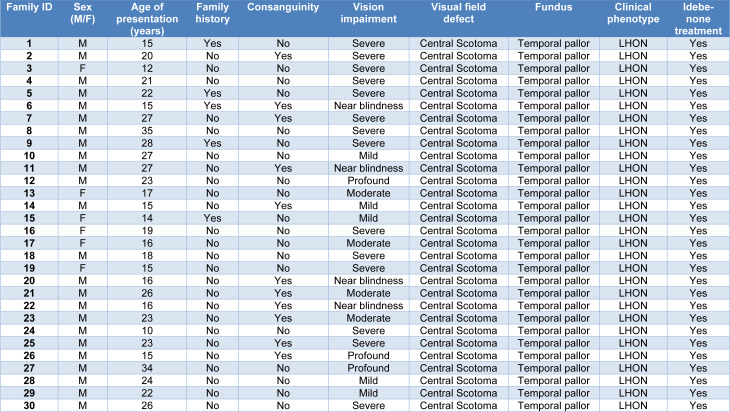
Ophthalmic clinical features of all LHON subjects

**Table 2:**
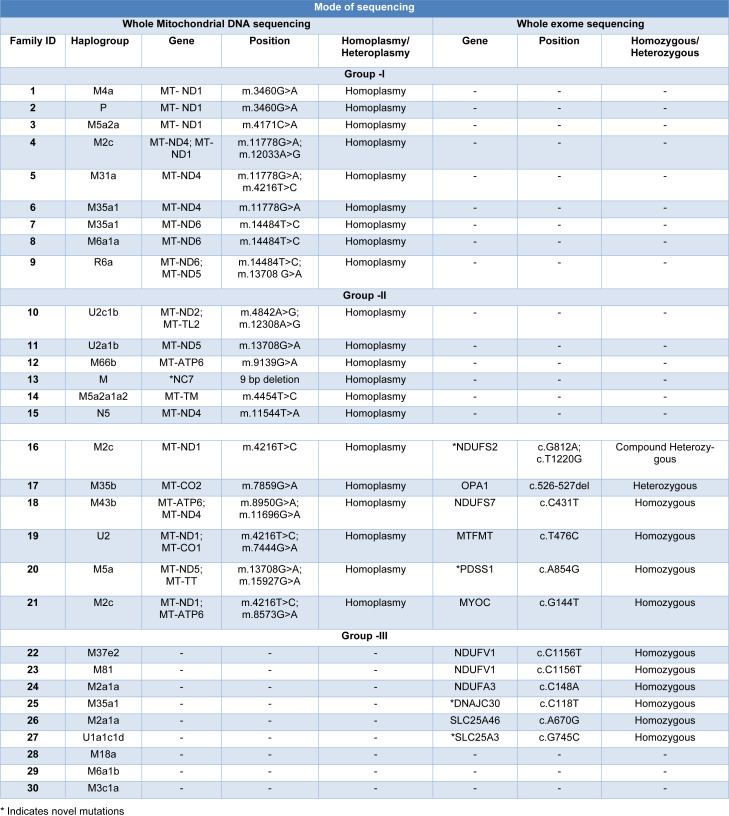
Summary of a list of pathogenic mito-nuclear genetic variants in LHON study subjects

**Figure 1:**
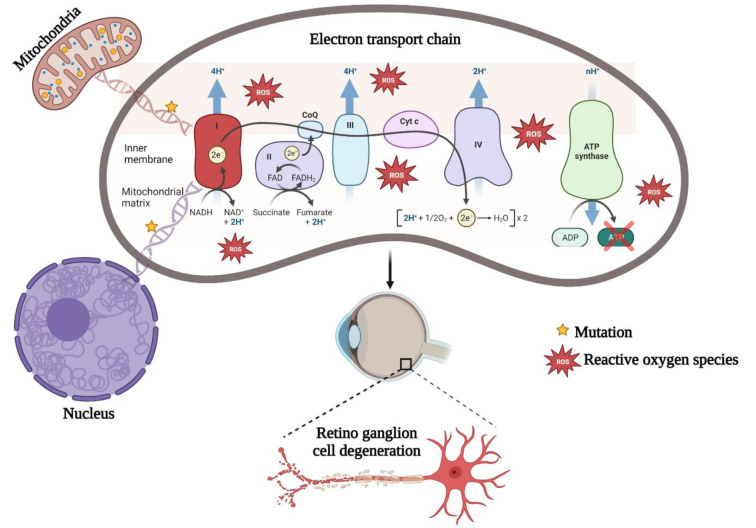
Graphical abstract
