# Supplementary material for: Exploring mito-nuclear genetic factors in Leber's hereditary optic neuropathy: insights from comprehensive profiling of unique cases
Source: EXCLI J. 2023 Oct 9;22:1077–91. doi: 10.17179/excli2023-6297 (PMC10694345; doi:10.17179/excli2023-6297)
Supplement: Supplementary information [file EXCLI-22-1077-s-001.pdf]

EXCLI Journal 2023;22:1077-1091 – ISSN 1611-2156
Received: July 03, 2023, accepted: October 06, 2023, published: October 09, 2023

Supplementary information to:

Original article:

EXPLORING MITO-NUCLEAR GENETIC FACTORS IN LEBER'S HEREDITARY OPTIC NEUROPATHY: INSIGHTS FROM COMPREHENSIVE PROFILING OF UNIQUE CASES

Prakash Chermakani[1,3], Poigaialwar Gowri[1,3], Shanmugam Mahesh Kumar[2], Periasamy Sundaresan[1,3*]

[1] Department of Molecular Genetics, Aravind Medical Research Foundation, Madurai, Tamil Nadu, India
[2] Neuro Ophthalmology Clinic, Aravind Eye Hospital, Madurai, Tamil Nadu, India
[3] Department of Molecular Biology, Aravind Medical Research Foundation - Affiliated to Alagappa University, Karaikudi, Tamil Nadu, India

[*] **Corresponding author:** Dr. Periasamy Sundaresan, Department of Molecular Genetics, Aravind Medical Research Foundation, 1, Anna Nagar, Madurai -625020, Tamil Nadu, India. Phone: +91-452-435 6100 (Extn: 423), E-mail: sundar@aravind.org

https://dx.doi.org/10.17179/excli2023-6297

This is an Open Access article distributed under the terms of the Creative Commons Attribution License (http://creativecommons.org/licenses/by/4.0/).

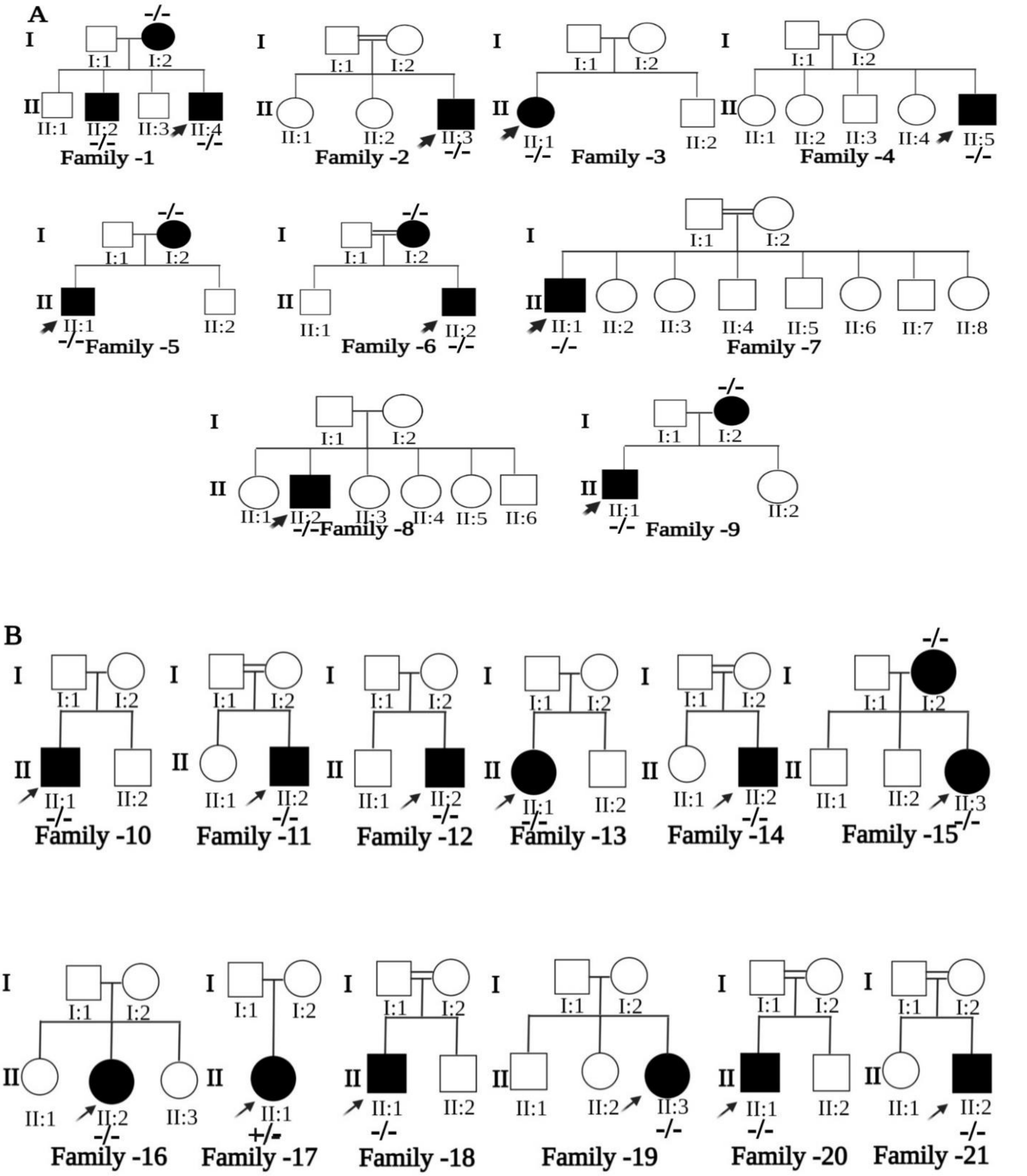
A
Family -1
Family -2
Family -3
Family -4
Family -5
Family -6
Family -7
Family -8
Family -9
B
Family -10
Family -11
Family -12
Family -13
Family -14
Family -15
Family -16
Family -17
Family -18
Family -19
Family -20
Family -21

EXCLI Journal 2023;22:1077-1091 – ISSN 1611-2156
Received: July 03, 2023, accepted: October 06, 2023, published: October 09, 2023

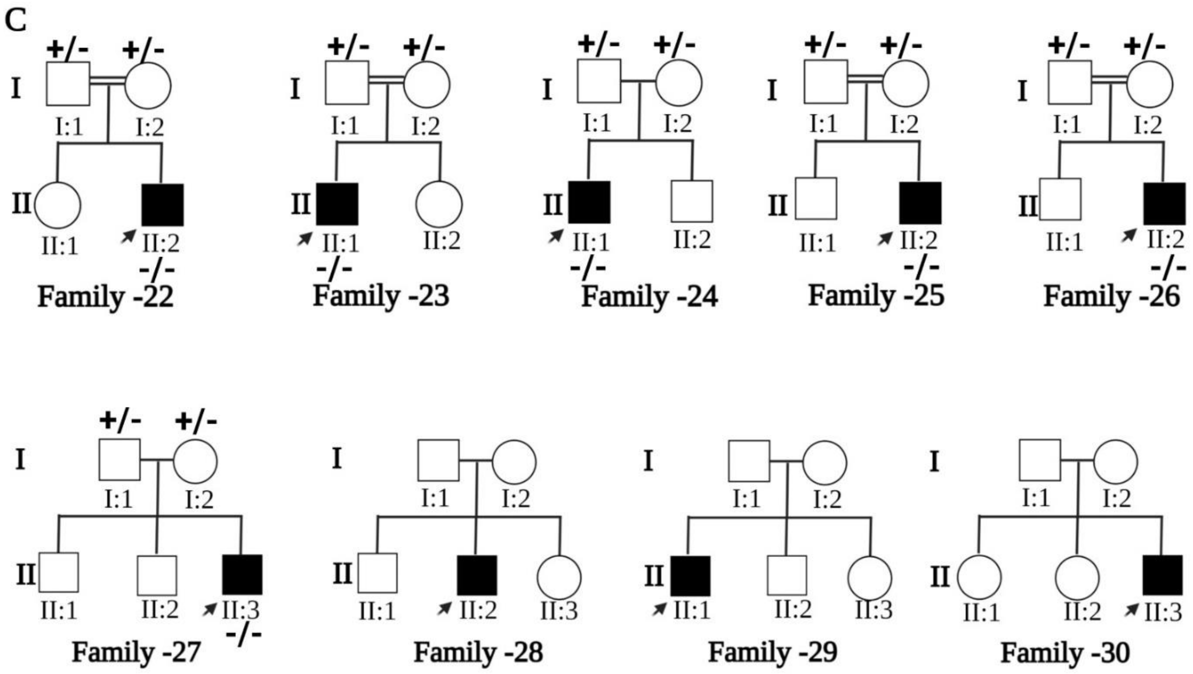

Supplementary Figure 1: Pedigree of thirty unrelated probands involved in this study. Solid symbols with an arrow represent proband. Consanguinity is represented by a double line. The genotype is denoted by (–/–) for homozygous variant carriers, (+/–) for heterozygous variant carriers. **A-** Group I; **B-** Group II and **C-** Group III

EXCLI Journal 2023;22:1077-1091 – ISSN 1611-2156
Received: July 03, 2023, accepted: October 06, 2023, published: October 09, 2023

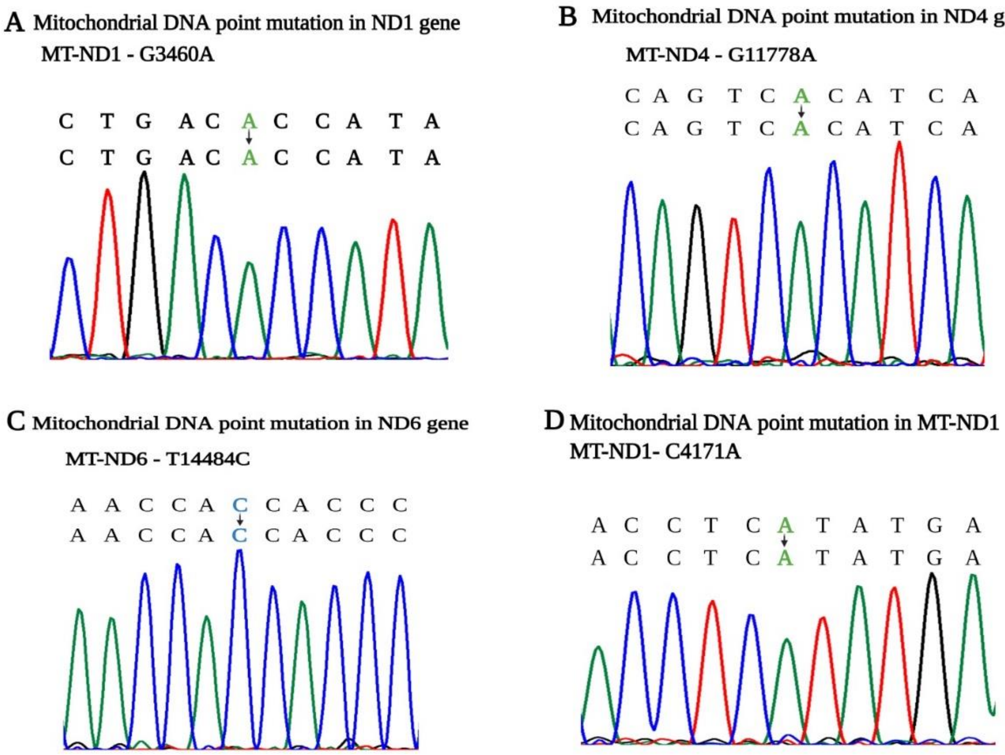

Supplementary Figure 2: Representative chromatogram of the probands in group I harboring primary mitochondrial DNA mutations (MT-ND1, MT-ND4 and MT-ND6 genes)

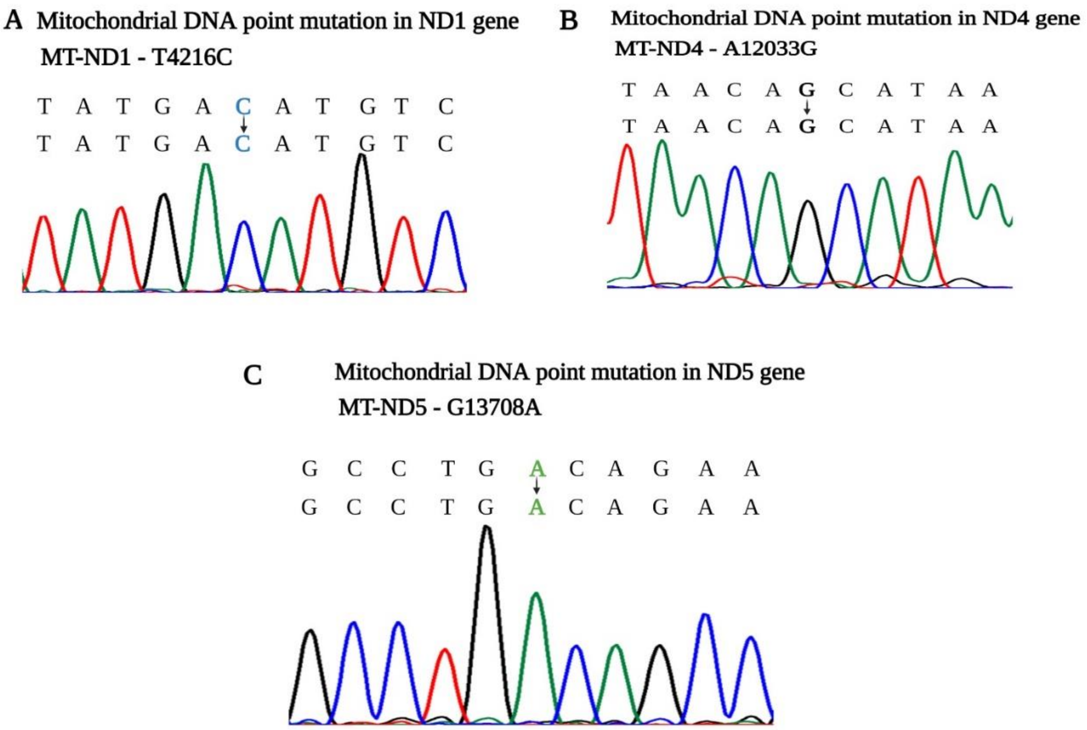

Supplementary Figure 2a: Sequencing chromatogram of the probands in group I harboring a co-occurrence secondary mitochondrial DNA mutations (MT-ND1, MT-ND4 and MT-ND5 genes)

EXCLI Journal 2023;22:1077-1091 – ISSN 1611-2156
Received: July 03, 2023, accepted: October 06, 2023, published: October 09, 2023

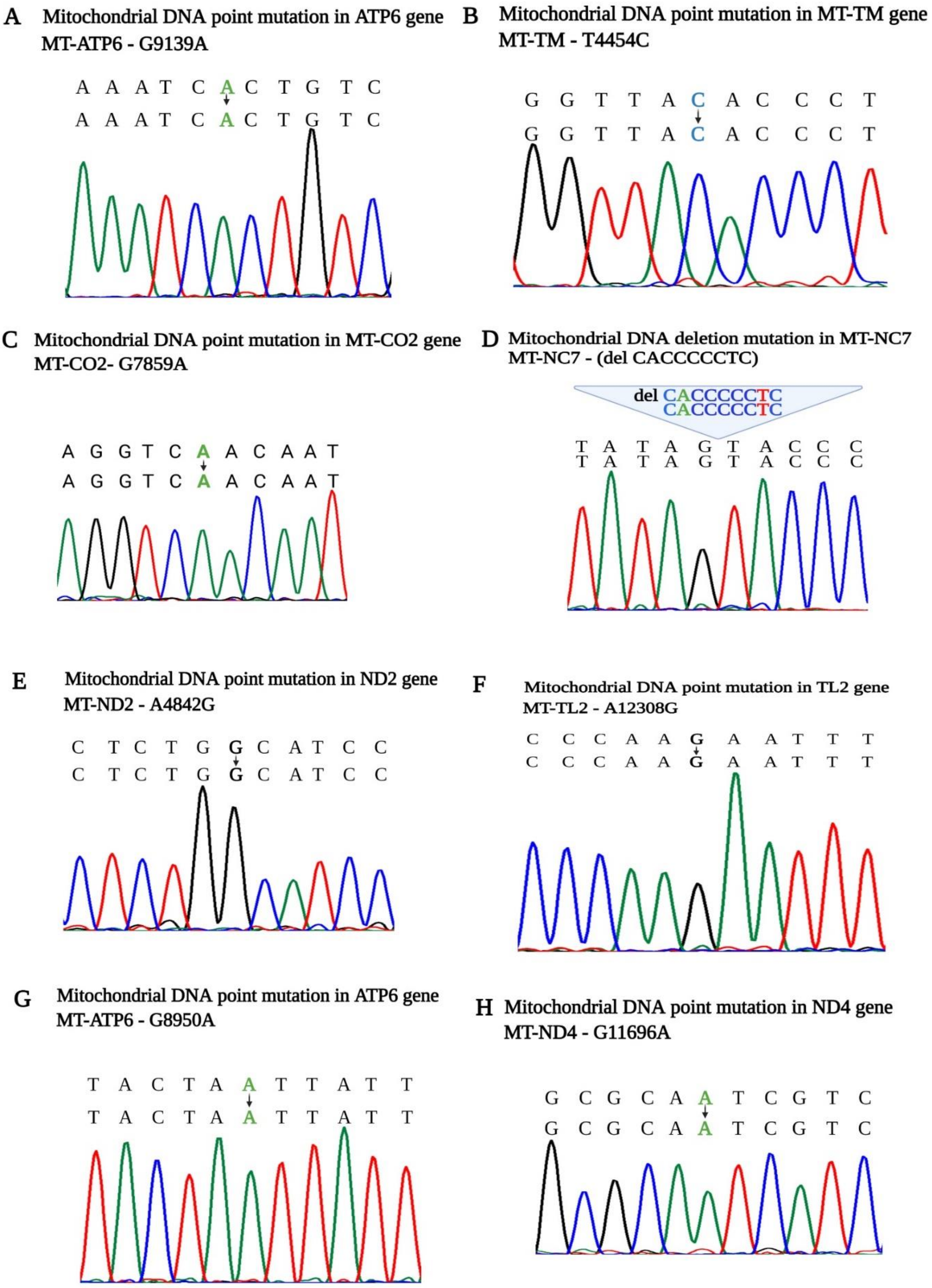

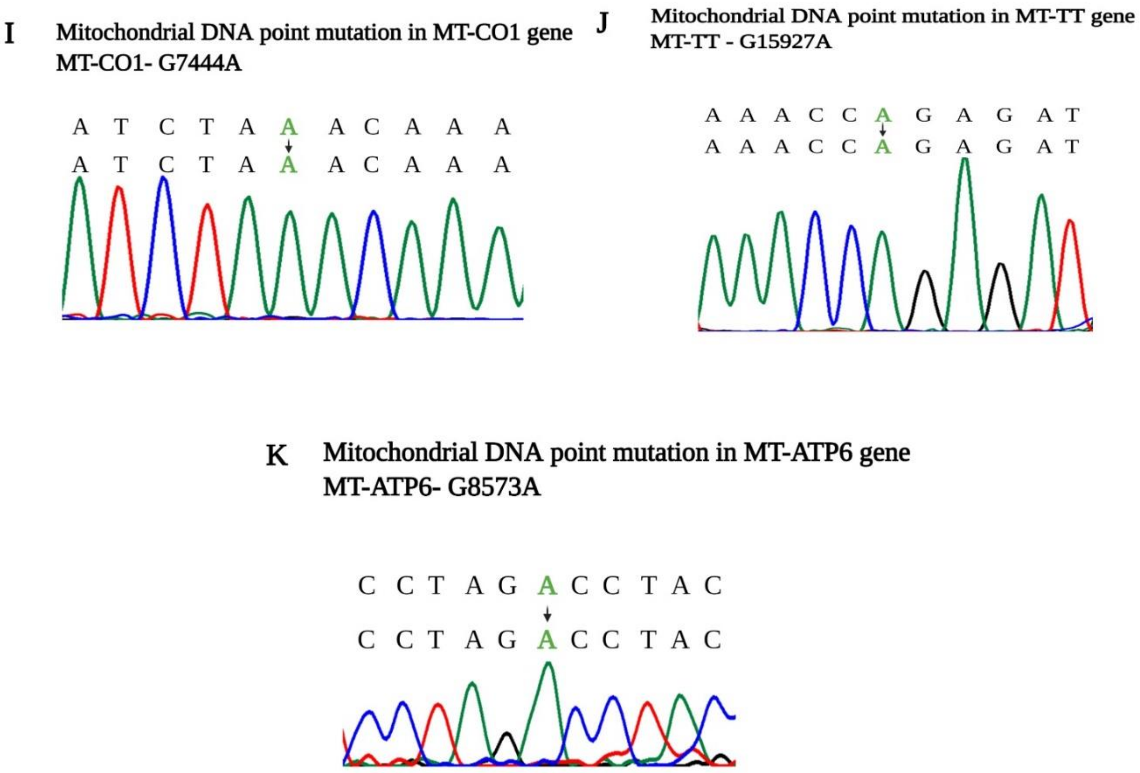

Supplementary Figure 3: Sequencing chromatogram from **A-K** represents the probands from group II harboring secondary mitochondrial DNA mutations.

EXCLI Journal 2023;22:1077-1091 – ISSN 1611-2156
Received: July 03, 2023, accepted: October 06, 2023, published: October 09, 2023

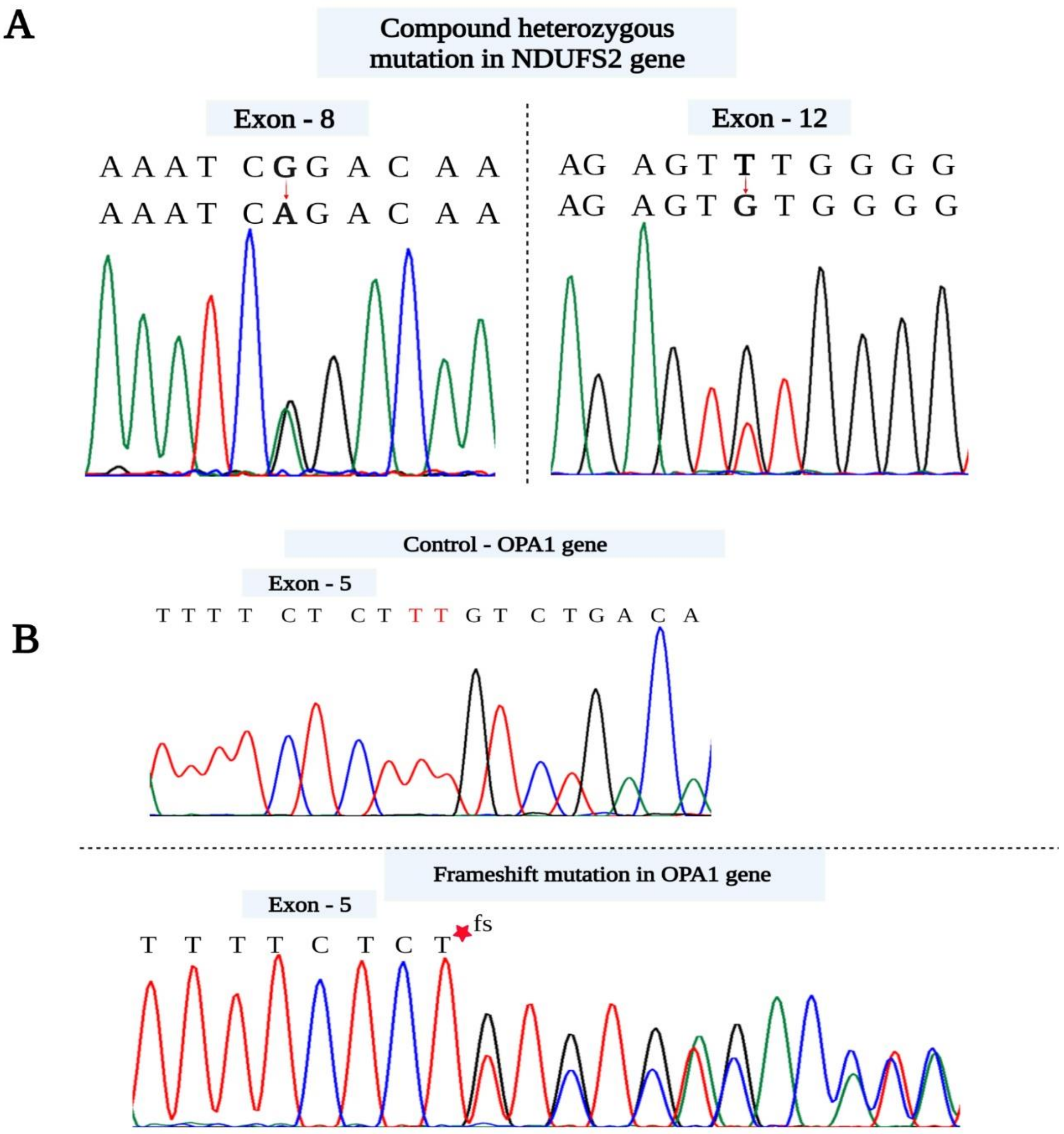

EXCLI Journal 2023;22:1077-1091 – ISSN 1611-2156
Received: July 03, 2023, accepted: October 06, 2023, published: October 09, 2023

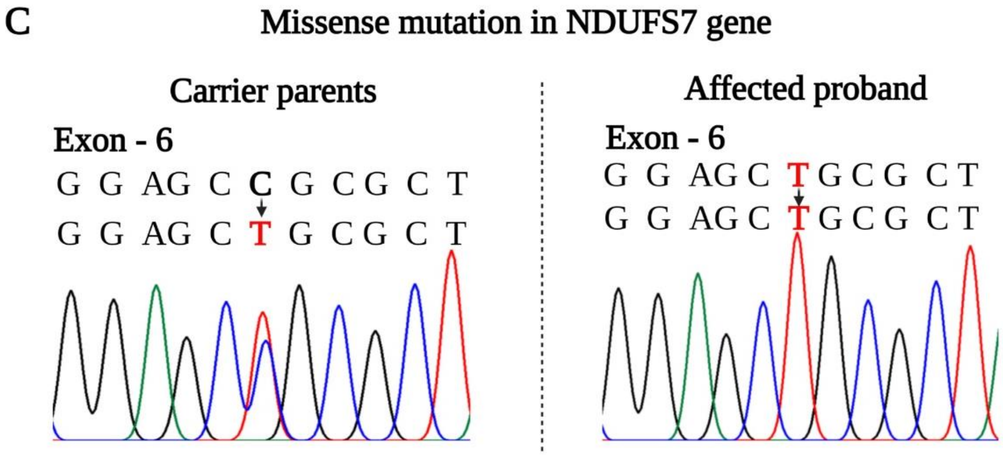

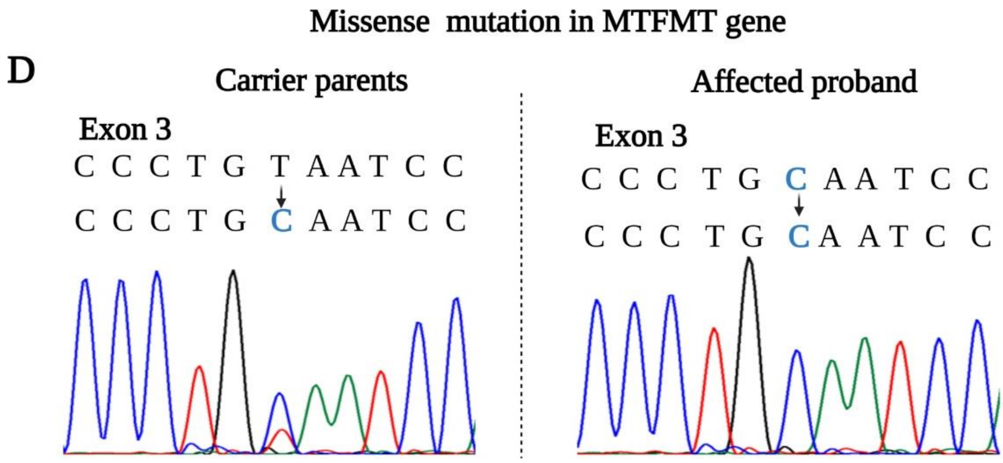

EXCLI Journal 2023;22:1077-1091 – ISSN 1611-2156
Received: July 03, 2023, accepted: October 06, 2023, published: October 09, 2023

E

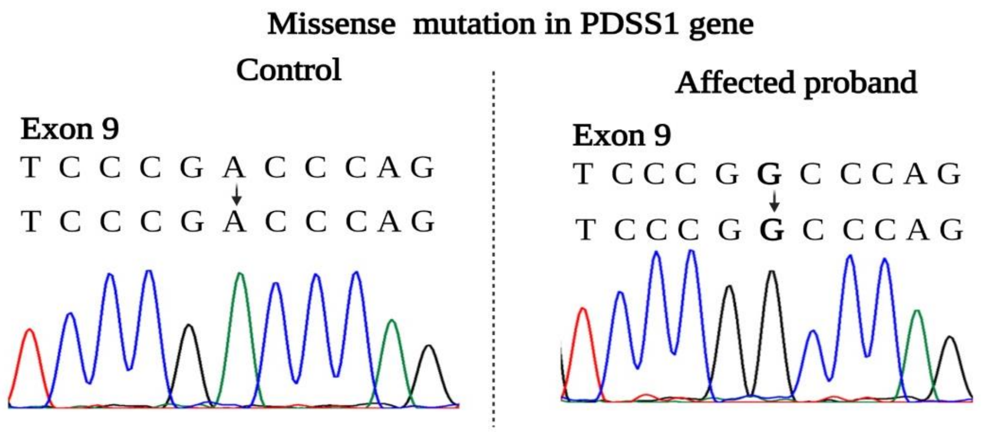

F

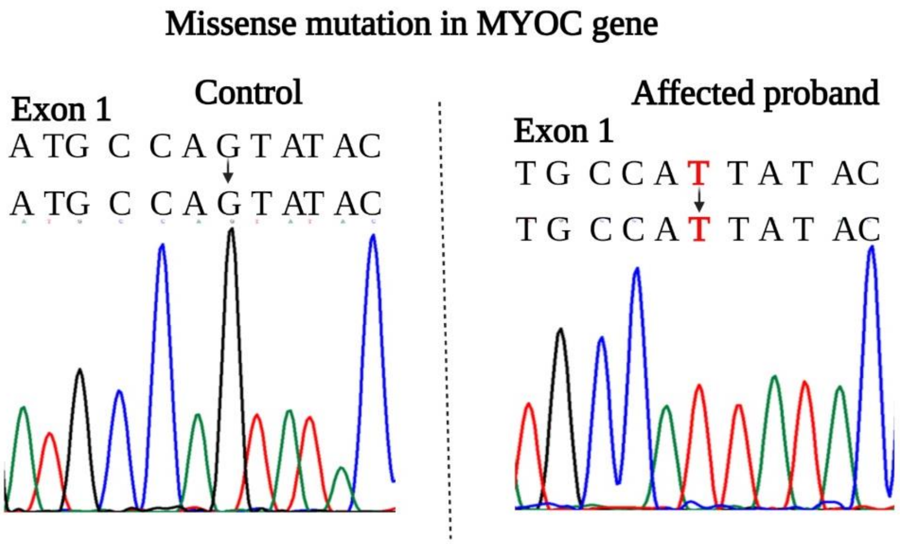

Supplementary Figure 4: Sequencing chromatogram from **A-F** represents the probands from group II harboring nuclear DNA mutations (NDUFS2, OPA1, NDUFS7, MTFMT, PDSS1 and MYOC gene)

EXCLI Journal 2023;22:1077-1091 – ISSN 1611-2156
Received: July 03, 2023, accepted: October 06, 2023, published: October 09, 2023

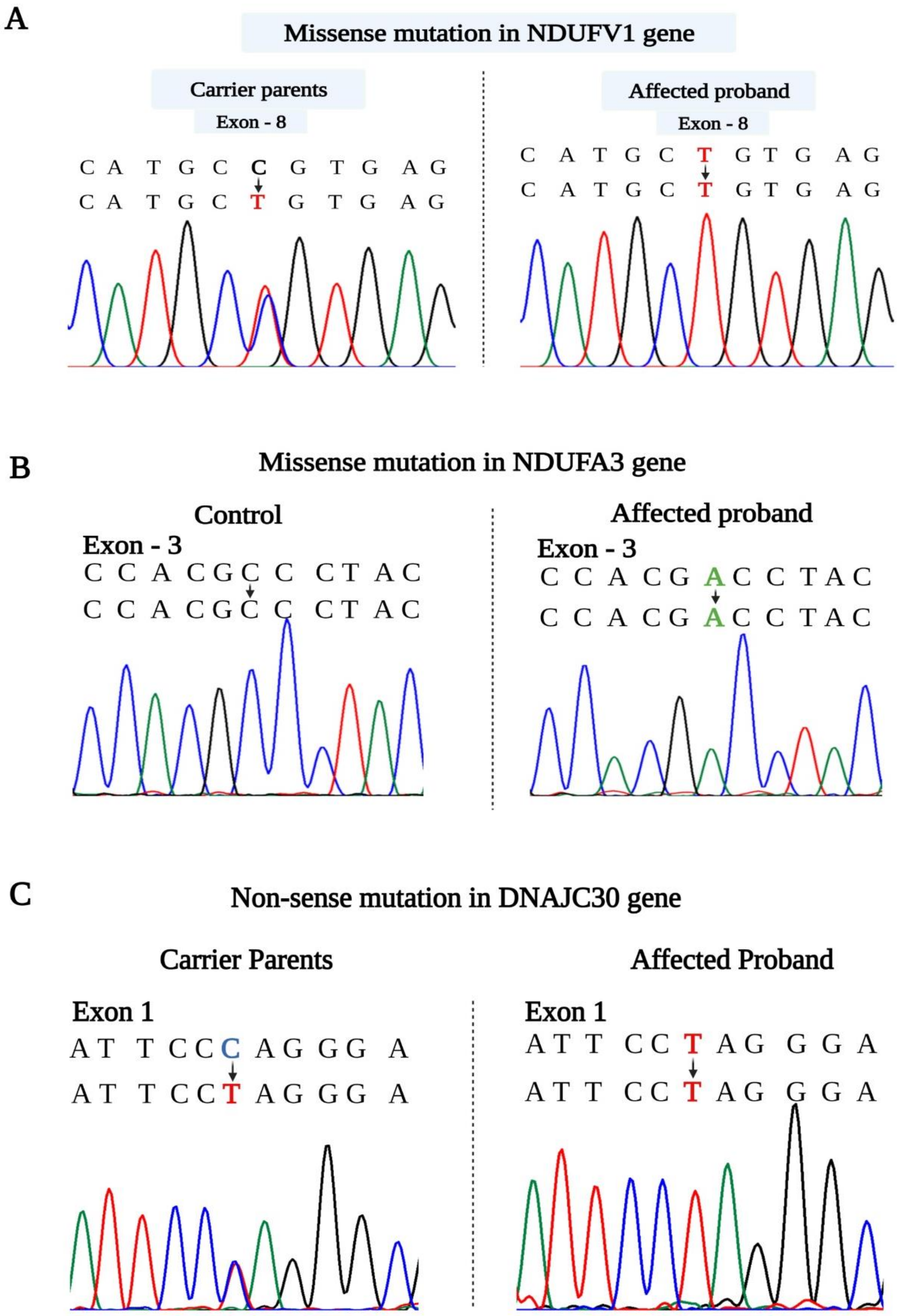

EXCLI Journal 2023;22:1077-1091 – ISSN 1611-2156
Received: July 03, 2023, accepted: October 06, 2023, published: October 09, 2023

D

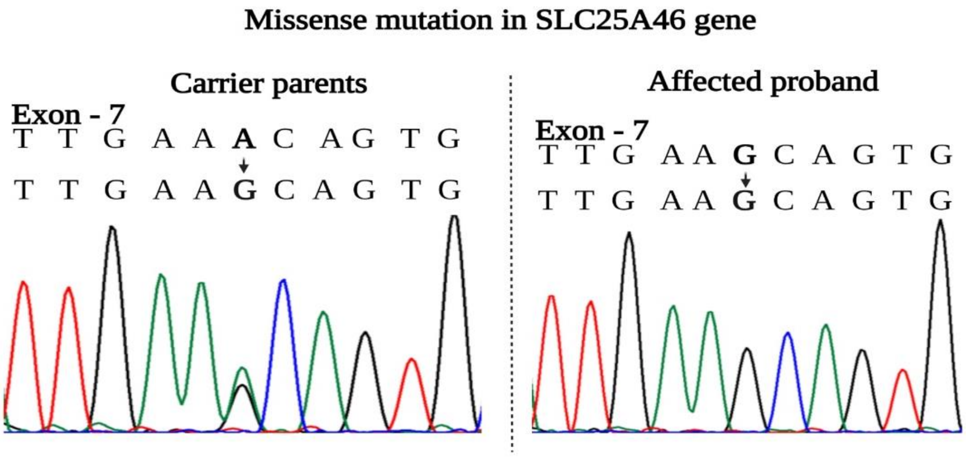

E

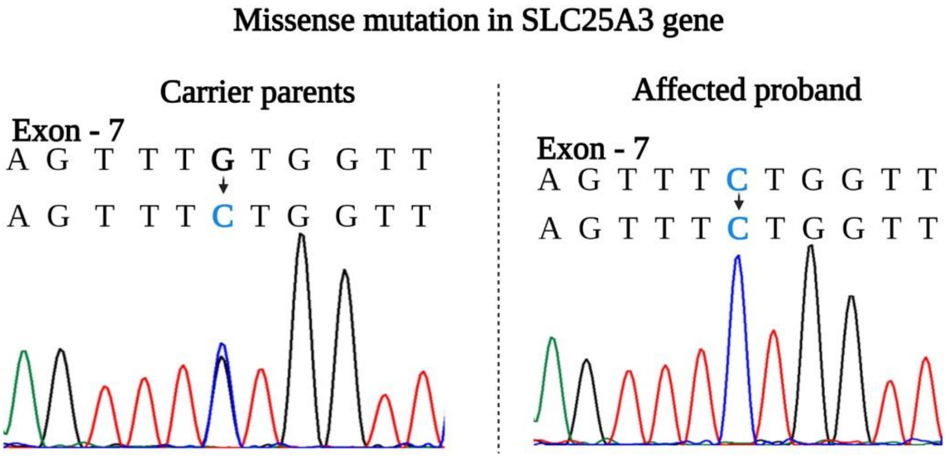

Supplementary Figure 5: Sequencing chromatogram from **A-E** represents the probands from group III harboring nuclear DNA mutations (NDUFV1, NDUFA3, DNAJC30, SLC25A46 and SLC25A3 gene

EXCLI Journal 2023;22:1077-1091 – ISSN 1611-2156
Received: July 03, 2023, accepted: October 06, 2023, published: October 09, 2023

Supplementary Table 1: Illustrates the key component of the sample group, family history and male to female ratio

Group	Probands	Total (Familial/ Sporadic)	Male (Familial/ Sporadic)	Female (Familial/ Sporadic)	Ratio (Male: Female)
	Total screened (n=30)	30 (5/25)	24 (4/20)	6 (1/5)	4:01
**Group I (1)**	LHON primary mutation (n=9)	9 (4/5)	8 (4/4)	1 (-/1)	8:01
**Group II (2)**	Secondary mitochondrial DNA mutation (n=12)	12 (1/11)	7 (-/7)	5 (1/4)	1.4:1
	*Secondary mitochondrial DNA mutation alone (n=6)	6(1/5)	4 (-/4)	2 (1/1)	2:01
	#Secondary mitochondrial DNA mutation + Nuclear gene mutation (n=6)	6 (-/6)	3 (-/3)	3 (-/3)	1:01
**Group III (no mtDNA mutation)**	Nuclear gene mutation (n=6)	6 (-/6)	6 (-/6)	0 (-/-)	NA
	No mutation (n=3)	3 (-/3)	3 (-/3)	0 (-/-)	NA

* indicates patients harbor only secondary mtDNA mutation
# indicates patients harbor both secondary mtDNA mutation and nuclear gene mutation
